# Shuangtengbitong tincture treatment of collagen-induced arthritis via downregulation of the expression of IL-6, IL-8, TNF-α and NF-κB

**DOI:** 10.3892/etm.2012.853

**Published:** 2012-12-05

**Authors:** KEDAN CHU, HAIYIN ZHENG, HUANG LI, YUQIN ZHANG, XUN ZHANG, WEI XU, LIDIAN CHEN

**Affiliations:** 1 Pharmacy College; Fujian University of Traditional Chinese Medicine, Fuzhou 350122, P.R. China; 2 Integrative Medicine College; Fujian University of Traditional Chinese Medicine, Fuzhou 350122, P.R. China; 3 Faculty of Rehabilitation Medicine, Fujian University of Traditional Chinese Medicine, Fuzhou 350122, P.R. China

**Keywords:** shuangtengbitong tincture, rheumatoid arthritis, interleukin-6, interleukin-8, tumor necrosis factor-α, nuclear transcription factor-κB

## Abstract

Rheumatoid arthritis (RA) is a chronic, systemic autoimmune disease and may lead to joint damage, synovial membrane destruction and cartilage and bone damage. RA is closely associated with increased expression of interleukin (IL)-6, IL-8, tumor necrosis factor-α (TNF-α) and nuclear transcription factor-κB (NF-κB). Therefore, inhibition of the expression of IL-6, IL-8, TNF-α and NF-κB is a promising strategy for the development of novel anti-RA therapies. The aim of this study was to investigate the effect of shuangtengbitong tincture (STBT) on the expression of IL-6, IL-8, TNF-α and NF-κB in synovial tissues of rats with collagen-induced arthritis (CIA). STBT as a clinical prescription created at Fujian University of Traditional Chinese Medicines (TCM) Affiliated People’s Hospital has been shown to be clinically effective in the treatment of RA. The model of Wistar rats with CIA was created using bovine type II collagen. The two treatment groups with CIA were administered STBT (1 ml per time) or Votalin (∼1 cm per time) for ∼1 month continuously. Following treatment, STBT suppressed paw swelling significantly (P<0.05) compared with the model group. STBT also improved pathological changes, STBT-treated rats showed a significant improvement in synovial hyperplasia, inflammatory infiltration, cartilage and bone destruction and other symptoms. The protein expression levels of IL-6, IL-8, TNF-α and NF-κB were markedly suppressed in synovial tissues of STBT-treated and Votalin-treated rats. Our findings demonstrate for the first time that STBT markedly reduces paw swelling, improves pathological changes and increases the expression of IL-6, IL-8, TNF-α and NF-κB in synovial tissues of CIA rats, which may partially explain the anti-RA activity of STBT.

## Introduction

Rheumatoid arthritis (RA) is a chronic, systemic autoimmune disease that may lead to joint damage, synovial membrane destruction, and cartilage and bone damage (1,2). Its prevalence varies between 0.5 and 1% worldwide (3). RA is an incurable disease that has a serious impact on human physical and mental health (4). Despite the prevalence of RA, its etiology and pathogenesis remain controversial. RA may be associated with immune regulation, infection, genetics, environment, neuro-psychological status and other factors (5). There are various mechanisms for the occurrence of RA in which joint inflammation and the autoimmune reaction play an important role.

Pro-inflammatory cytokines, including interleukin (IL)-6, IL-8 and tumor necrosis factor (TNF)-α, are important cytokines in inflammation and are considered to be the most important mediators involved in the pathogenesis of RA (6). Previous studies have also discussed the role of cytokines in RA (6,7). TNF-α has been shown to play an important role in RA (8–10) and is known to mediate a variety of effector functions relevant to the pathogenesis of RA. In addition, therapies targeting TNF-α are also recognized as effective treatments for patients with RA. Proinflammatory cytokine IL-6 is an abundantly expressed cytokine in rheumatoid synovium which is considered to lead to joint damage (11) and chemotactic activity (12). The chemokine IL-8 may activate neutrophils and enhance the phagocytosis of neutrophils and the activity of their lysosomal enzyme. IL-8 has a chemotactic effect on basophils and T cells. A number of of these cytokines are not randomly present, but their expression is controlled by a network or hierarchy which induces nuclear transcription factor (NF)-κB expression. Therefore downregulation of pro-inflammatory cytokines may be an effective and rational approach for the treatment of RA.

Shuangtengbitong tincture (STBT) is a clinical prescription of Fujian University of Traditional Chinese Medicines (TCM) Affiliated People’s Hospital and is officially recorded in the Min drug system (Z20111008) for therapy of RA. STBT consists of four components, namely *Tripterygium wilfordii* Hook f., *Caulis sinomenii*, *Dioscorea nipponica* Makino and *Glycyrrhiza uralensis* Fisch. These components have the ability to dispel wind, eliminate dampness, dredge meridians, reduce swelling and relieve pain. It has been shown that STBT is effective against RA. However, no studies on the anti-inflammatory activity of STBT have been performed. In the present study, a CIA model was used to investigate the effects of STBT on acute arthritis, chronic joint damage and anti-inflammatory activity.

## Materials and methods

### Materials

A Dionex Ultimate 3000 liquid chromatograph equipped with DAD detector was bought from Dionex Ltd. [Thermo Fisher Scientific (Shanghai) Co. Ltd, China]; a KQ-500DE ultrasonic clearing machine was purchased from Kunshan Ultrasonic Instrument Co., Ltd. (Kunshan, China); a XS105 electronic analytical balance was supplied by Mettler-Toledo Instruments (Shanghai) Co., Ltd., (Shanghai, China); a YLS-7B paw plethysmometer was obtained from Yi Yan Technology Development Co., Ltd. (Jinan, China). Complete Freund’s adjuvant (CFA) and type II collagen were purchased from Sigma Chemical (St. Louis, MO, USA). Antibodies were bought from Cell Signaling (Beverly, MA, USA). All other chemicals used, unless otherwise stated, were obtained from commercial sources.

### Animals

A total of 40 SPF Wistar male rats provided by the Animal Care and Use Committee of Fujian University of TCM, weighing 180–220 g, were purchased from Shanghai Slac Laboratory Animal Co. Ltd. (Shanghai, China, license number 200700518360). The animals were housed under controlled temperatures (21–23°C), relative humidity 55±5%, a 12-h light/dark cycle and had free access to a standard rat diet and water. All animal experiments were conducted in accordance with international ethical guidelines and the National Institutes of Health Guide concerning the Care and Use of Laboratory Animals.

### Preparation of STBT

**Figure 1.**
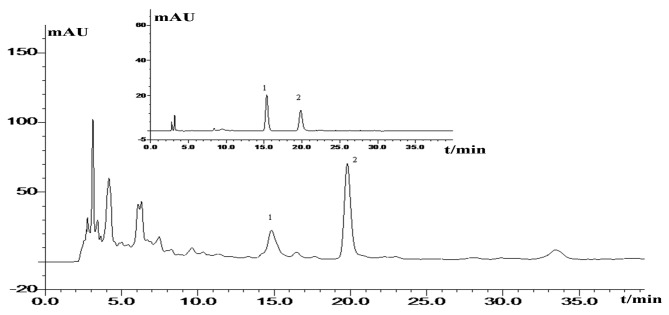
HPLC of target analytes in the STBT sample. Inset: HPLC of target analytes standard mixture. In liquid chromatograms 1 is triptolide and 2 is sinomenine. STBT, shuangtengbitong tincture; HPLC, high-performance liquid chromatography.

STBT is a mixture of four herbs that were bought from Fujian University of TCM Affiliated People’s Hospital. The herbs were verified by Professor Wei Lu from the pharmacy college of Fujian University of TCM. The herb voucher specimens were deposited in the pharmacy college of Fujian University of TCM. STBT was prepared as the preparation of STBT hospital prescription of Fujian University of TCM Affiliated People’s Hospital. *Tripterygium wilfordii* Hook f., *Caulis sinomenii*, *Dioscorea nipponica* Makino and *Glycyrrhiza uralensis* Fisch. were crushed into coarse powder, mixed and then steeped 10 times in 80% ethanol. After 48 h, extraction was percolated slowly, collected and filtrated. Finally the solution was adjusted to 10 g/ml (original medicinal materials/volume). The prepared STBT was processed according to our preliminary study (13) and then was filtrated through 0.45-μm microporous membranes prior to analysis by liquid chromatography. The major peaks were identified as the marker compounds (Fig. 1).

### Induction of CIA model and treatments

A total of 40 Wistar rats were randomly assigned to five experimental groups with ten animals in each: normal, model, Votalin ointment and STBT groups. With the exception of the normal group, the other groups were used to create the CIA model. First, 2 mg/ml collagen was emulsified with an equal volume of CFA to a final concentration of 0.1 mg/ml. Second, 2 ml collagen emulsion was injected into the base of the tail of each rat via intradermal injection. After 7 days, a provocation test was performed; 1 ml collagen emulsion was injected into the base of the tail of each rat via intradermal injection. Drugs were administered intragastrically twice a day from the first day after primary immunization. In the STBT group, STBT at a dose of 1 ml per time was sprayed onto the back of shaved rats. The area of back treated was 3×3 cm, according to the manufacturer’s instructions. The Votalin ointment group were administered Votalin ointment at a dose of ∼1 cm per time as described above. The normal group and model group were not treated. Animals were medicated for 35 days continuously.

### Swelling dimension observation

A YLS-7B plethysmometer was employed to measure the volume of the hind paws of rats (14). The paw volumes before and after modeling were recorded. The volume of each rat paw was measured every 3 days from the 7th day following the primary immunization.

### Histopathological assessment

On the final day of treatment, all rats were anesthetized and the right hind knee was removed and fixed in 4% neutral-buffered formalin for 24 h. After decalcifying with 12.5% EDTA (pH 7.0) for ∼20 days, the right hind knee was paraffin-embedded, sectioned at a 5-mm thickness and stained with hematoxylin and eosin.

### Immunohistochemistry (IHC) analysis

The above paraffin sections were used for IL-6, IL-8, NF-κB and TNF-α immunohistochemical analysis (15) according to the manufacturer’s instructions (USCN Life Science Inc., Houston, TX, USA). Five high-power fields (×400) were selected in each slide randomly and the average proportion of positive cells in each field were counted by the true color multi-functional cell image analysis management system (Image-Pro Plus, Media Cybernetics, Inc., Rockville, MD, USA). The average proportion of positive cells in each field was counted and classified into 5 grades: no staining was scored as 0, 1–10% of cells stained scored as 1, 11–50% as 2, 51–80% as 3 and 81–100% as 4. Staining intensity was rated on a scale of 0 to 3, with 0, negative; 1, weak; 2, moderate; and 3, strong. The raw data were converted into the IHS by multiplying the quantity and staining intensity scores (16).

### RNA extraction and RT-PCR analysis

Total RNA was extracted from synovial tissue with TRIzol reagent. Oligo(dT)-primed RNA was reverse transcribed to cDNA. The obtained cDNA was used to determine the mRNA levels of TNF-α and NF-κB by PCR with *Taq* DNA polymerase (Fermentas Life Science, Beijing, China) according to the manufacturer’s instructions. The sequences of NF-κB and TNF-α used for amplification were as follows: NF-κB forward, 5′-GCG CAT CCA GAC CAA CAA TAA C-3′ and reverse, 5′-GCC GAA GCT GCA TGG ACA CT-3′; TNF-α forward, 5′-CTCCCA GGT TCT CTT CAA GG-3′ and reverse, 5′-TGG AAG ACT CCT CCC AGG TA-3′. The conditions for PCR were set at the following: initial denaturation at 95°C for 3 min, 30 sec to denature at 95°C, 72°C for 45 sec to extend and 35 cycles for amplification. The annealing temperatures were 60°C. Glyceraldehyde-3-phosphate dehydrogenase (GAPDH) was employed as a normalization control. Finally, PCR products were analyzed by gel electrophoresis (1.5% agarose) and the DNA bands were examined in a Gel Documentation System (Model Gel Doc 2000; Bio-Rad, Hercules, CA, USA).

### Statistical methods

Data are expressed as mean ± standard deviation (SD). SPSS 16.0 statistical software (SPSS Inc., Chicago, IL, USA) was used for statistical analysis. The comparisons between groups were performed using the Student’s t-test. P<0.05 was considered to indicate a statistically significant result.

## Results

### Swelling dimension

From the 7th to the 31st day, the changes in paw swelling are displayed in Table I. On approximately the 10th day, the knee joints of the rats began to swell. With time, joint and paw swelling became more severe. Paw and knee joint swelling volume reached a maximum on approximately the 18th day in the model group. The groups treated with Votalin ointment and STBT demonstrated inhibition and were significantly different (P<0.05 and P<0.01, respectively) to the model group. STBT suppressed paw swelling at the start and produced a greater inhibition effect than Votalin ointment.

### Histopathological changes

**Figure 2.**
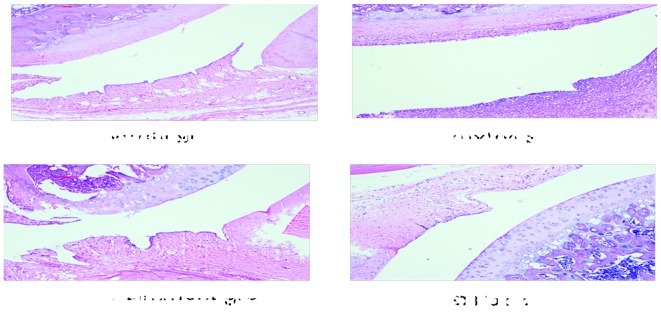
Histological changes in CIA rats. Representative lesions using HE stain of hind knee joint from rats are shown (magnification, ×100). CIA, collagen-induced arthritis; HE, hematoxylin and eosin; STBT, shuangtengbitong tincture.

Representative histopathological lesions in the hind knee joint from normal, model, Votalin ointment and STBT groups are shown in Fig. 2. Synovial hyperplasia, disorganized arrangement, infiltration of inflammatory cells, cartilage destruction and bone destruction were observed in the model group. Histopathological lesions were ameliorated in the treated groups to a different extent. The histopathological lesions of the STBT group improved well. It is shown that only moderate proliferation of the synovium occurred, cell morphology was regularly arranged, a small number of inflammatory cells infiltrated, the surface of the cartilage was smooth and there were no clear signs of bone erosion in the CIA rats of the STBT group.

### Protein and mRNA expression of IL-6, IL-8, TNF-α and NF-κB

**Figure 3.**
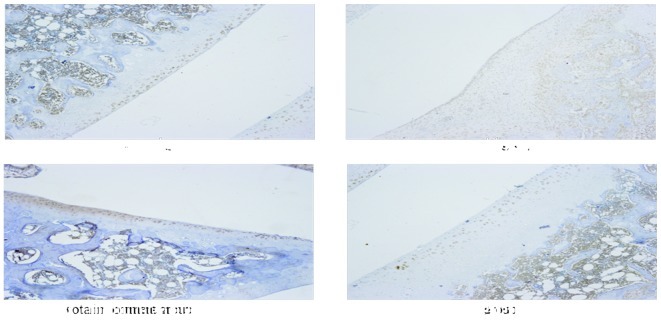
Immunohistochemical determination of protein expression. Representative images of staining for TNF-α are shown (magnification, ×10). TNF, tumor necrosis factor; STBT, shuangtengbitong tincture.

**Figure 4.**
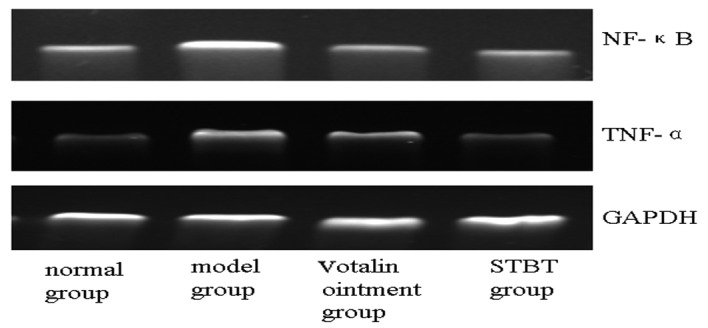
Determination of mRNA expression of TNF-α and NF-κB. The levels of TNF-α and NF-κB in synovial tissues were determined by RT-PCR analysis. GAPDH was used as the internal control. TNF-α, tumor necrosis factor-α; NF-κB, nuclear transcription factor-κB; GAPDH, glyceraldehyde-3-phosphate dehydrogenase.

The protein and mRNA expression were determined by IHC analysis and RT-PCR. A semi-quantitative score was calculated according to positive cell ratio and staining intensity. IHS is shown in Table II. The cytoplasm of positive cells were brown, representative IHC pictures of TNF-α are shown in Fig. 3. Positive areas of IL-6, IL-8, NF-κB and TNF-α were significantly enlarged in the model group compared with the normal group, the difference was statistically significant (P<0.01). In the STBT group, expression of IL-6, IL-8, NF-κB, TNF-α was weaker compared with the model group (P<0.05). The results of the RT-PCR (Fig. 4) showed that the mRNA expression of TNF-α and NF-κB in the model group was significantly increased, compared with that in the normal group (P<0.05), however, NF-κB and TNF-α were downregulated by the treatment with Votalin ointment or STBT. Data from RT-PCR showed that the pattern of mRNA expression of TNF-α and NF-κB was similar to their respective protein levels.

## Discussion

RA is a chronic and systemic autoimmune disease which is characterized by arthrosynovitis; its main clinical manifestations are multi-joint synovitis and articular damage (17,18). CIA has been proved to share various immunological and pathological features with human RA (18,19). CIA has become one of the most widely used models for screening new drugs for the treatment of rheumatoid diseases. CIA rats were used to demonstrate the anti-inflammatory effect of STBT in the current study.

The herbs in STBT have been used for hundreds of years and their efficacy is documented. The STBT formula is chemically complex with hundreds of components. Certain bioactive chemicals have been acknowledged, including: triptolide and tripterine in *Tripterygium wilfordii* Hook f. (20–22), sinomenine in *Caulis sinomenii*(23,24), dioscin in *Dioscorea nipponica* Makino (25) and glycyrrhizic acid and Liquiritin in *Glycyrrhiza uralensis* Fisch. (26). Each component of STBT may play an important role in the treatment of RA. However, numerous herbs are often used together under the theories of TCM. It is believed that the interactions among these herbs contribute more to the anti-RA effect of STBT than their effects alone.

In the present study, it was observed that the swelling dimensions of the group treated with STBT decreased as compared with the model group. In addition, according to the histopathological change of ankle joint analysis, it was suggested that STBT was able to alleviate the hyperplasia of the synovial membrane, bone destruction and other symptoms. These results are consistent with those of a previous study (27). STBT possesses certain anti-inflammatory effects.

Pro-inflammatory cytokines located in synovium, including IL-1, IL-6, IL-8 and TNF-α, have been known to play a major role in the progression of joint destruction (6,19). IL-1 and TNF-α promote the induction of adhesion molecules and proteinase gene expression, and play a major role in the progression of joint destruction and proliferation of synoviocytes, and also increase the production of IL-6, IL-8 and other chemokines (28). A number of these cytokines were not randomly present but formed a network or hierarchy which controlled their expression. The molecular basis of destruction in RA or CIA remains to be elucidated. The present study showed that STBT has an inhibitory action on expression levels of IL-6, IL-8, TNF-α and NF-κB, as revealed by IHC analysis and RT-PCR. It has been reported that the efficacy of IL-6, IL-8 and TNF-α blockade has been shown by their specific inhibitors (29).

The transcription regulator NF-κB is an important nuclear transcription factor in RA. NF-κB plays a pivotal role at the stages of initiation and perpetuation of inflammation in disease. NF-κB regulates the expression of numerous types of cytokines and proinflammatory mediators (30). Our results for the expression level of NF-κB were in agreement with those reported in the literature (23,2). The suppression of NF-κB may be responsible for the decrease of levels of IL-6, IL-8 and TNF-α in synovial tissue.

We observed that STBT has a therapeutic effect on CIA and the effect may correlate with the suppressive effect on the production of pro-inflammatory cytokines and NF-κB.

## Figures and Tables

**Table I.** Changes in paw swelling.

Group	Paw volume before in ammation (ml)	Paw swelling after in ammation at different times (ml)								

		7 days	10 days	13 days	16 days	19 days	22 days	25 days	28 days	31 days
Control	1.58±0.07	1.59±0.07	1.59±0.05	1.61±0.08	1.60±0.06	1.63±0.07	1.63±0.08	1.64±0.05	1.66±0.04	1.66±0.07
Model	1.59±0.01	1.62±0.10	1.72±0.09^b^	1.80±0.13^b^	2.31±0.22^b^	2.96±0.25^b^	3.01±0.17^b^	2.99±0.14^b^	2.99±0.14^b^	2.87±0.23^b^
Votalin ointment	1.60±0.05	1.62±0.06	1.69±0.07^c^	1.75±0.11^c^	2.22±0.13^d^	2.60±0.13^d^	2.63±0.16^d^	2.15±0.41^d^	2.00±0.16^d^	1.98±0.14^d^
STBT	1.58±0.06	1.62±0.03	1.67±0.08^c^	1.72±0.03^d^	2.02±0.21^d^	2.39±0.17^d^	2.57±0.04^d^	2.02±0.14^d^	1.92±0.14^d^	1.84±0.24^d^

Values are presented as mean ± SD.

a P <0.05,

b P <0.01 compared with the control group;

c P <0.05,

d P <0.01 compared with the model group. STBT, shuangtengbitong tincture.

**Table II.** Comparison of immunohistochemical scores of IL-6, IL-8, TNF-α and NF-κB in synovial tissue.

Group	IL-6	IL-8	TNF-α	NF-κB
Normal	0	0	0	0
Model	6.87±2.02^b^	6.03±1.07^b^	6.35±2.58^b^	6.60±2.60^b^
Votalin ointment	5.83±0.95^c^	4.80±2.03^c^	5.65±1.64^c^	5.93±2.53
STBT	5.80±1.30^c^	5.07±1.56^c^	5.51±2.03^c^	5.71±0.96^c^

Values are presented as mean ± SD.

a P<0.05,

b P<0.01 compared with the normal group;

c P<0.05,

d P<0.01 compared with the model group. STBT, shuangtengbitong tincture; IL, interleukin; TNF-α, tumor necrosis factor-α; NF-κB, nuclear factor-κB.

## Abbreviations:

RA: rheumatoid arthritis
STBT: shuangtengbitong tincture
CIA: collagen-induced arthritis
IL-6: interleukin-6
IL-8: interleukin-8
TN F-α: tumor necrosis factor-α
NF-κB: nuclear transcription factor κB
IHC: immunohistochemstry
IHS: immunohistochemical score
